# Reliability and Discriminant Ability of an Instrumented Timed Up and Go Test in People With Postsurgical Orthopedic Conditions: Quantitative Study

**DOI:** 10.2196/82632

**Published:** 2026-04-01

**Authors:** Marica Giardini, Ilaria Arcolin, Valerio Antonio Arcobelli, Michela Picardi, Sabato Mellone, Marco Godi

**Affiliations:** 1 Department of Physical Medicine and Rehabilitation, Institute of Veruno, Istituti Clinici Scientifici Maugeri IRCCS, Gattico-Veruno, Italy; 2 Department of Electrical, Electronic, and Information Engineering–Guglielmo Marconi (DEI), University of Bologna, Viale del Risorgimento 2, Bologna, 40136, Italy; 3 Department of Neurorehabilitation Sciences, Casa di Cura Igea, Milano, Italy

**Keywords:** instrumented Timed Up and Go, iTUG test, inertial measurement unit, IMU, reliability, orthopedic patient, wearable sensor

## Abstract

**Background:**

The Timed Up and Go (TUG) test is widely used to assess mobility and fall risk in older adults and orthopedic patients. Its instrumented variant (iTUG), based on inertial measurement units, enables an objective quantification of motor performance and can even be implemented using smartphone technology. However, its broader clinical adoption remains limited by concerns about reliability, feasibility, and the interpretability of the extracted parameters.

**Objective:**

This study aimed to evaluate the test-retest reliability of variables derived from a single-sensor iTUG in orthopedic inpatients undergoing rehabilitation and to determine whether a subset of reliable sensor-based metrics can support a multidimensional assessment of functional mobility and discriminate among common orthopedic conditions.

**Methods:**

We recruited 104 inpatients at discharge from a rehabilitation ward after total hip arthroplasty, total knee arthroplasty, or femur fracture. Each participant performed the iTUG test on 2 consecutive days using a smartphone-based solution consisting of an inertial measurement unit placed on the lower back. From 100 extracted variables, those with excellent test-retest reliability (intraclass correlation coefficient ≥0.75) were retained. Exploratory factor analysis was used to identify underlying mobility domains, and linear discriminant analysis with 10-fold cross-validation tested their ability to classify diagnostic groups.

**Results:**

Out of 100 iTUG-derived variables, 36 demonstrated excellent test-retest reliability, and 25 were retained for multivariate analysis. Exploratory factor analysis identified 5 factors—walking ability, pace or rhythm, sit-to-walk smoothness, turning ability, and mediolateral angular stability—explaining 80.8% of the total variance. These factors showed good classification accuracy (68%) and achieved an area under the curve of 0.86 and an overall mean accuracy of 0.68 (SD 0.14) for distinguishing among total hip arthroplasty, total knee arthroplasty, and femur fracture. In contrast, total iTUG duration alone yielded an area under the curve of 0.62. All patients used walking aids, and gait variables were more reliable than jerk-based or coordination metrics.

**Conclusions:**

The single-sensor iTUG provides reliable and clinically informative metrics that go beyond traditional stopwatch timing, enabling a multidimensional view of functional mobility in orthopedic patients. The approach is feasible, interpretable, and compatible with real-world mobile health apps, supporting personalized rehabilitation monitoring and future integration into digital decision support systems.

## Introduction

The Timed Up and Go (TUG) test is widely recognized for its simplicity and clinical applicability [1]. It is considered a fundamental tool for assessing functional mobility, including elements of balance, walking ability, and transitional movements in older adults [2-4]. The test involves rising from a chair, walking 3 m, performing a 180° turn, returning to the chair, and sitting down, with the total time recorded using a stopwatch [5].

Its widespread clinical use stems from its practicality and robust clinimetric properties. Within neurological practice, the TUG test is useful in identifying fall risk among people with conditions such as stroke and Parkinson disease [6]. In orthopedics, the test has been recommended for evaluating patients with hip fractures [7] and for distinguishing between future recurrent fallers and nonfallers in frail older adults post hip fracture [8]. Additionally, the TUG has been demonstrated as a valuable test in predicting recovery trajectories: shorter recovery stays in patients with total hip arthroplasty (THA) [9] and early identification of postsurgical rehabilitation needs in patients with total knee arthroplasty (TKA) [10].

The advent of inertial measurement units (IMUs) has expanded the TUG’s potential through detailed and objective kinematic analysis of its subcomponents. The instrumented TUG (iTUG) enables the detection of specific functional impairments [11], improves fall risk sensitivity [12], supports rehabilitation monitoring, and predicts clinical outcomes [13-15].

Indeed, a recent review has examined different methods for analyzing the iTUG, focusing on specific aspects such as key variables, clinical applications, and predictive value [1]. While this review underscores the clinical potential of the iTUG, its psychometric properties remain insufficiently explored. Existing research has primarily addressed the reliability of parameters in healthy older adults or individuals with neurological conditions [11,16,17].

To the best of our knowledge, no studies have analyzed iTUG reliability in patients with orthopedic conditions. This study aimed to evaluate the test-retest reliability of the iTUG in orthopedic inpatients in a rehabilitative setting. Specifically, we targeted 3 common orthopedic conditions: THA, TKA, and femur fractures. Additionally, we sought to identify the most reliable iTUG variables and assess their ability to discriminate between these patient groups using exploratory factor analysis (EFA) and linear discriminant analysis (LDA). We hypothesize that iTUG-derived variables can effectively distinguish between the 3 orthopedic groups and that domains related to dynamic mobility tasks, such as walking, will provide the highest discriminative value.

The objective is not to enable the diagnosis of those orthopedic conditions; rather, the hypothesis is that, based on measures derived from the iTUG, it may be possible to discriminate among these conditions in the phases immediately prior to discharge, given the distinct biomechanical implications underlying each. This initial evidence would support the use of the iTUG as a promising tool for patient follow-up, whether at home or during subsequent check-up visits.

## Methods

### Patients

Between April 2023 and December 2023, all patients admitted to the musculoskeletal ward of the Department of Physical Medicine and Rehabilitation at the Istituti Clinici Scientifici Maugeri Istituto di Ricovero e Cura a Carattere Scientifico (Gattico-Veruno, Piedmont, Italy) for rehabilitation following orthopedic surgery were screened for inclusion during the last 3 to 4 days of hospitalization. Patients were eligible if their primary admitting diagnosis was unilateral THA, TKA, or a femur fracture surgically repaired with internal fixation. Inclusion criteria included being aged 18 years or older, the ability to walk at least 10 meters without assistance (Functional Ambulation Category score ≥3), using the walking aid used in the final days of hospitalization, and the cognitive ability to understand instructions (Functional Independence Measure cognitive subscale ≥29).

Exclusion criteria included the presence of major associated injuries of the lower or upper limbs, contraindications to mobilization and weight-bearing on the lower extremities, clinical instability, disorders of bone metabolism other than osteoporosis, neurological or major cardiac diseases, pathological fractures, a prior history of overt dementia, terminal illness (life expectancy <6 mo), and prehospitalization dependence on nursing home care or inability to walk independently.

### Ethical Considerations

The study was approved by the local ethics committee (2678 CE) and was conducted in accordance with the World Medical Association’s Declaration of Helsinki. All participants provided informed consent for study participation. To ensure participants’ privacy and confidentiality, all data were anonymized and coded using unique identification numbers. No personally identifiable information was stored in the research database or in the investigational application, and access to the data was restricted to authorized study personnel. Participants did not receive any financial compensation for their participation.

### Setting and Procedures

#### iTUG Test

All evaluations were conducted in the Laboratory of Posture and Movement at the ICS Maugeri of Veruno. The laboratory provided a spacious, quiet area with adequate lighting, suitable for performing iTUG tests. The iTUG test was recorded following established protocols developed by Caronni et al [18,19]. During the test, participants wore a commercial IMU secured with a belt around their lower back at the level of the third lumbar vertebra (L3).

All participants were instructed to perform the iTUG test, which consisted of standing up from a chair, walking 3 m, pivoting, returning to the chair, and sitting down. An ordinary chair with a seat height of 44 cm was used. To mark the turning point, a yellow and black tape was placed on the floor at a distance of 3 m from the chair.

Participants initiated the task upon receiving a start signal from the iTUG commercial software and were instructed to maintain a comfortable and safe walking pace throughout the test.

#### Instrumentation

The iTUG system was supplied by mHealth Technologies srl (Monte San Pietro, Italy) in a portable briefcase containing the following components: a motion sensor with its charging station and power supply; 2 fabric belts of different sizes, equipped with Velcro closures, to secure the sensor to the patient; an Android smartphone with its charging cable; a user manual; a quick guide; and a declaration of conformity. The motion sensor is a lightweight IMU (22 g) with dimensions of 54 mm×33 mm×14 mm. It incorporates a triaxial accelerometer, a triaxial gyroscope, and a triaxial magnetometer, offering a maximum sampling frequency of 200 Hz. The sensor is positioned at the third lumbar vertebra (L3) using a Velcro-secured belt, with the buttons facing downward. Proper positioning is critical, as incorrect placement results in unreliable data.

The system connects the IMU to a smartphone via Bluetooth, with the smartphone acting as the central processing and control unit. During the iTUG test, the IMU captures movement signals, which are processed by the smartphone app “mTUG,” developed by mHealth Technologies srl. The app manages the system and generates a report on the patient’s kinematic performance during the test. The IMU signals provide the total duration of the TUG test and, through validated algorithms [16,17], automatically segment the test into four distinct phases: (1) sit-to-walk, (2) walk, (3) turn 180°, and (4) turn-to-sit. From each test, a total of 100 metrics is obtained (Multimedia Appendix 1).

#### Procedure

The iTUG procedures were conducted by 4 licensed physical therapists who underwent a dedicated half-day training session focused on standardized test administration, including sensor placement, task instructions, and use of the data acquisition and analysis software. Each participant was evaluated twice: first on the third-to-last day of hospitalization and then 24 hours later. A rater, randomly selected from the pool of 4 trained raters, conducted the evaluation. The rater provided instructions, demonstrated the procedure, secured the belt with the sensor, and initiated the measurement by activating the software to give the start signal. To ensure familiarity with the test, participants performed an initial trial that was not recorded and excluded from the analysis, following established guidelines [11]. After the familiarization trial, participants completed 2 recorded trials. During each trial, the rater manually recorded the time to complete the TUG test using a stopwatch in addition to the automated measurements provided by the iTUG system. The entire procedure, including the familiarization trial and the 2 recorded trials, was repeated 24 hours later by a different randomly selected trained rater.

### Data Analysis

#### Reliability Measures

The best iTUG trial, that is, the trial with the shortest time recorded by the stopwatch, recorded on the first day was compared with the best trial recorded on the second day. These iTUG tests were used to calculate the test-retest reliability of the 100 variables collected for each participant, expressed as intraclass correlation coefficients (ICCs). In particular, we used an ICC 2,1 (model 2, form 1) [20] because the rater was not fixed and the data used to calculate the ICC were the measures of a single walking trial. An ICC less than 0.40 was considered to indicate poor reliability, between 0.40 and 0.59 indicated moderate reliability, between 0.60 and 0.74 indicated good reliability, and 0.75 or greater indicated excellent reliability [21]. All variables that obtained an ICC value less than 0.75 during reliability evaluation were excluded from the subsequent analysis.

Only after completing the variable reduction process, and thus after analyzing the correlation matrix, was it decided to calculate other measures of agreement for the resulting variables. Indeed, the SE of measurement (SEM), to assess the absolute error of the instrument, and the minimum detectable change (MDC), which represents the smallest change considered significant beyond the measurement error of an individual, were employed [20,22]. The SEM was calculated by multiplying the SD of the measurements by the square root of 1 minus the ICC (SD × √1-ICC). The MDC at 90% was derived using the formula $MDC=1.65\times \sqrt{2}\times SEM$.

#### Correlation Matrix

For simplicity, we chose to perform all analyses on the variables collected during the first trial on the first day of testing. The variables included in the correlation matrix were tested for normal distribution using the Shapiro-Wilk test and Q-Q plot. For variables with nonnormal distributions, a logarithmic transformation was applied, followed by a reassessment of normality. Subsequent analyses were performed using the transformed variables where appropriate.

The data were screened for factorability using several well-described criteria, including the Kaiser-Meyer-Olkin (KMO) measure for sampling adequacy, the Bartlett test of sphericity, an anti-image correlation matrix [23-26], and the Pearson correlation and determinant score.

- The KMO statistic ranges from 0 to 1, with values above 0.50 considered acceptable for individual variables and a total value above 0.70 deemed adequate for the overall correlation matrix [20].
- The Bartlett test of sphericity evaluates the null hypothesis that the original correlation matrix is equal to an identity matrix (one with all 1’s on the diagonal). A significant Bartlett test (*P*<.05) suggests that the variables are suitable for factor analysis. This condition prevents the application of factor analysis because it seeks items that are sufficiently correlated for the extraction of the factors or dimensions [20].
- The anti-image correlation matrix was inspected to test whether partial correlations among variables were small; we calculated the percentage of elements with values exceeding 0.30.
- From the correlation matrix, created to explore the relationships between variables, only variables with Pearson correlation coefficients ≥0.20 but <0.90 were considered, in order to avoid the presence of both isolated variables and multicollinearity [20].
- The determinant of a correlation matrix assesses issues related to extreme multicollinearity (ie, questionnaire items with high correlations) and singularity (questionnaire items with perfect correlations). These issues can hinder the application of exploratory factor analysis, indicating the need to exclude problematic items. Since the determinant of a correlation matrix can range from 0 to 1, it should be greater than 0.00001 to mitigate the potential problems associated with multicollinearity and singularity [27].

#### Exploratory Factor Analysis

We conducted an EFA using principal component extraction with varimax rotation. While principal component analysis is not inherently a latent variable model, it is commonly employed in EFA as a factor extraction method when the objective is to identify underlying constructs [23]. The resulting factors were interpreted as latent dimensions of iTUG performance. iTUG variables were first standardized and mean-centered. The number of factors to retain was determined using the following standard criteria [28]:

- Kaiser criterion [28,29], which retains only factors with eigenvalues ≥1
- Cattell scree test [30], based on the visual inspection of the plot of eigenvalues
- Horn parallel analysis [31], where random eigenvalues are compared to the observed data to identify meaningful factors

Only variables with factor loadings ≥|0.40| were considered relevant contributors to each factor. The factor scores for each individual were then computed and used in subsequent analyses, representing the extent to which each participant expressed the corresponding latent dimension [32].

#### Linear Discriminant Analysis

The factor scores derived from the EFA were entered into an LDA with 10-fold cross-validation to evaluate the ability of iTUG variables to classify patients into the 3 diagnostic groups (THA, TKA, and femur fracture). Model performance was assessed using receiver operating characteristic (ROC) analysis, and discrimination was quantified using the area under the curve (AUC). The AUC of the proposed model was compared with that obtained from manually timed TUG performance (stopwatch) and with AUC values derived from an LDA model, including all iTUG variables with good reliability (ICC>0.75). AUC values were computed and reported separately for the THA, TKA, and femur fracture groups.

AUC values were interpreted as follows: poor discrimination, AUC<0.70; moderate discrimination, AUC between 0.70 and 0.89; and high discrimination, AUC≥0.90. Classification accuracy, calculated from the confusion matrix, was also reported separately for each diagnostic group.

#### Statistical Analysis

Mean (SD) values were used for descriptive statistics, and mean (SE) values were used for the figures. One-way ANOVA was used for the direct comparison between 2 variables (“Total Duration till initial contact with the chair” vs TUG test duration by chronometer). The level of significance was set at *P*<.05. Statistical analysis was conducted using R software (R Foundation for Statistical Computing), with the packages *blandr*, *EFAtools*, *FactoMineR*, *graphics*, *irr*, *lattice*, *MASS*, *methods*, *multiUS*, *psych*, and *stats*.

#### Sample Size Calculation

The sample size required for the reliability study was calculated based on the ICC method [33]. With 2 assessors using the iTUG system and assuming a reliability of at least 0.7, a sample of 104 patients was determined to be adequate.

## Results

### Participants

Of the 266 inpatients screened, 112 (42%) met the inclusion criteria and were enrolled in the study. The remaining patients were excluded from the study for the following reasons: no walking ability, cognitive impairments, clinical instability, other types of fractures besides femur fractures, or severe comorbidities. Among the 112 recruited participants, 3 did not undergo the second-day trial due to clinical reasons, 2 did not return for reassessment, and for 3 others, iTUG data could not be extracted due to software malfunctions. Thus, the final sample comprised 104 participants. Of these, 47% were hospitalized for femur fractures, 36% for THA, and 17% for TKA. Table 1 presents the clinical characteristics of the assessed patients.

**Table 1.** Characteristics of the recruited sample (N=104).

Characteristics	Value
Demographic	
Age (y), mean (SD)	73.6 (10.9)
Sex, n	
Female	69
Male	35
Clinical	
BMI, mean (SD)	22.8 (3.5)
Interval between surgery and assessment (d), mean (SD)	26.4 (6.8)
Injury side, n	
Right	54
Left	50
Type of surgical intervention, n	
TKA^a^	18
THA^b^	37
Femur fracture by osteosynthesis	20
Femur fracture by THA	29

a TKA: total knee arthroplasty.

b THA: total hip arthroplasty.

### Reliability Measures

**Figure 1.**
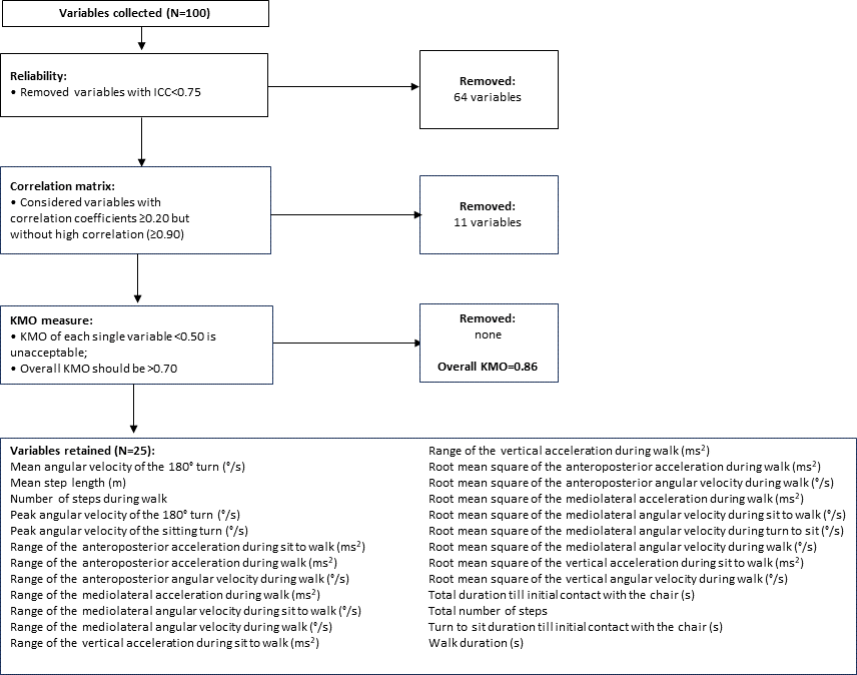
Flowchart describing the initial selection of variables from the instrumented Timed Up and Go (iTUG) test. ICC: intraclass correlation coefficient; KMO: Kaiser-Meyer-Olkin.

The total iTUG duration recorded using the stopwatch was highly comparable to the iTUG-derived variable “Total Duration till initial contact with the chair” (correlation: y = 0.99x + 0.24; *R*²=0.99). The mean time was 17.5 (SD 6.3) seconds for manual versus 17.6 (SD 6.3) seconds for iTUG, with no significant differences among orthopedic groups (ANOVA; *P*=.09). The ICC for this variable was 0.90 (95% CI 0.849‐0.928), and it was therefore included in the subsequent EFA. Figure 1 illustrates the stepwise selection process for reliable iTUG variables.

**Figure 2.**
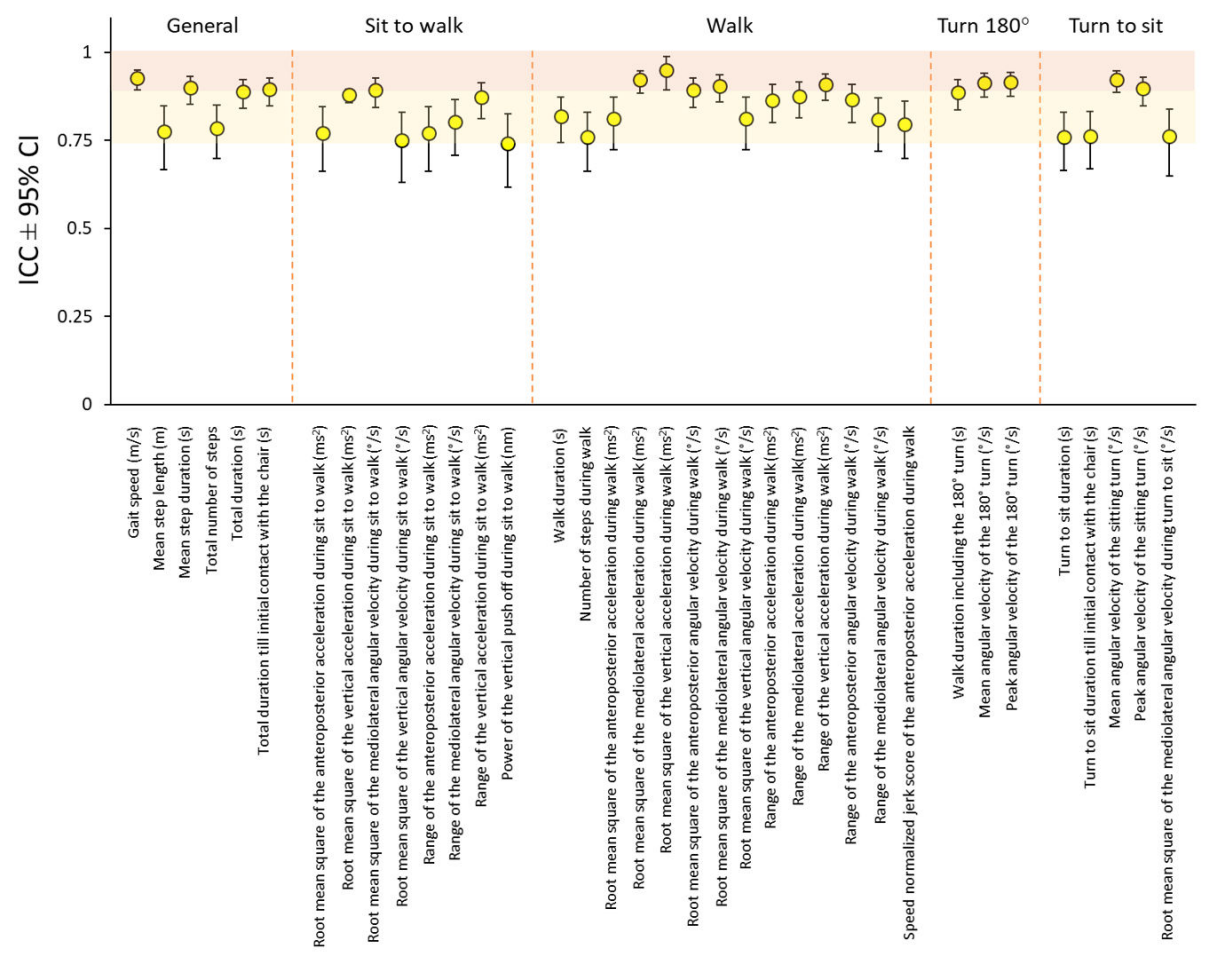
Representation of the test-retest reliability of the 36 variables, divided into the different tasks of the instrumented Timed Up and Go (iTUG) test, with intraclass correlation coefficient (ICC) values >0.75. The top orange bar indicates excellent ICCs, meaning >0.90, while the yellow bar indicates ICCs >0.75, used as a cutoff to identify highly reliable variables.

ICC values for all 100 iTUG variables initially considered were calculated. Subsequently, only variables with high ICC values were retained, resulting in a total of 36 variables. Figure 2 illustrates the ICC of these variables, providing an assessment of test-retest reliability: each point represents the ICC value for a variable, with error bars indicating the 95% CIs around the estimate. These intervals reflect the precision of the ICC measurement; narrower intervals denote higher confidence in the reliability estimate. With these 36 reliable variables, a correlation matrix was calculated.

### Correlation Matrix and Factor Analysis

**Figure 3.**
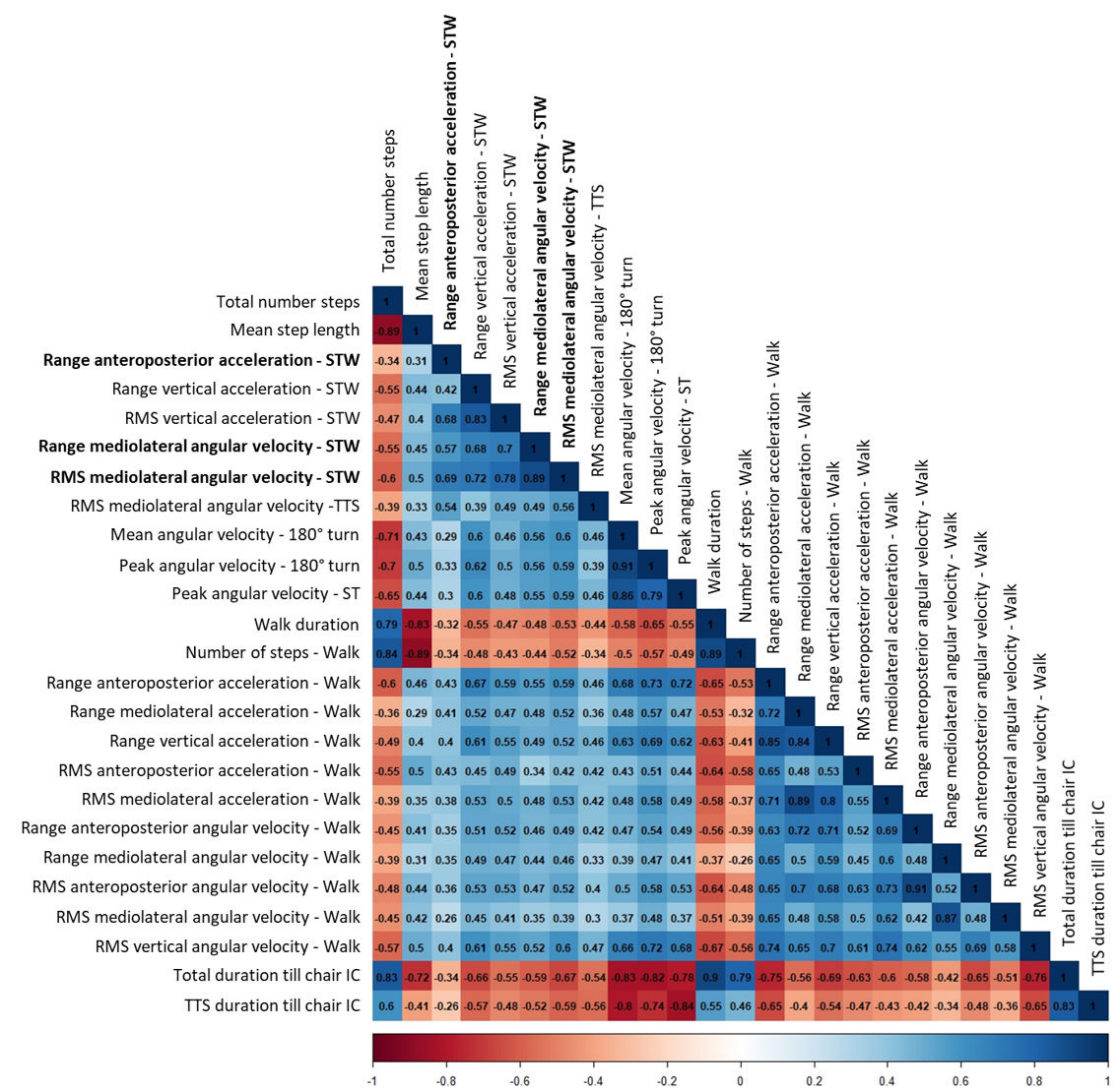
Correlation matrix, shown as heatmap, between the final 25 variables of the instrumented Timed Up and Go (iTUG) test. Data from all participants were used to prepare the matrix (N=104). Variables shown in bold were already normally distributed and did not require logarithmic transformation. IC: initial contact; RMS: root mean square; ST: sitting turn; STW: sit-to-walk; TTS: turn-to-sit.

Of the 36 retained variables, 6 were normally distributed; the remaining variables were log-transformed, and their distributions were then evaluated again for normality. Based on the predefined criteria for the correlation matrix (see “Methods” section), 11 variables were eliminated from the initial group of 36 variables (Figure 3).

Sampling adequacy was confirmed (KMO=0.86), and the Bartlett test indicated significant correlations among variables (*χ*²_300_=3199.92; *P*<.001). The determinant of the correlation matrix was 7.36 × 10^-16^, indicating no issues with multicollinearity or singularity. The percentage of partial correlations below the threshold of 0.3 in the anti-image correlation matrix was 16%, a value that can be considered adequate for proceeding with factor analysis.

Table 2 shows the correlations among the 25 selected variables, with all pairwise correlations exceeding the minimum acceptable threshold of 0.2. The table also reports the factor loadings for each variable on the 5 extracted factors, which were labeled according to their conceptual meaning and the naming convention proposed by Coni et al [34]: “walking ability,” “pace or rhythm,” “sit-to-walk smoothness,” “turning ability,” and “mediolateral angular stability.” The factor loadings were derived from a PCA with varimax rotation, which preserves orthogonality and retains the full variance of the original data. PCA identified 5 components with eigenvalues ≥1 (Kaiser criterion), and this was confirmed by the Horn parallel analysis. Together, these 5 components explained 80.8% of the total variance in the iTUG dataset, with individual contributions of 0.54, 0.09, 0.07, 0.06, and 0.05 of the variance, respectively.

**Table 2.** Factor loadings of the instrumented Timed Up and Go (iTUG) variables after principal component analysis^a^.

	Walking ability	Pace or rhythm	Sit-to-walk smoothness	Turning ability	Mediolateral angular stability
Range of the anteroposterior acceleration during walk (ms^2^)	*0.529*	—^b^	—	0.489	0.410
Range of the anteroposterior angular velocity during walk (°/s)	*0.811*	—	—	—	—
Range of the mediolateral acceleration during walk (ms^2^)	*0.828*	—	—	—	—
Range of the vertical acceleration during walk (ms^2^)	*0.704*	—	—	0.403	—
Root mean square of the anteroposterior acceleration during walk (ms^2^)	*0.474*	0.492	—	—	—
Root mean square of the anteroposterior angular velocity during walk (°/s)	*0.791*	—	—	—	—
Root mean square of the mediolateral acceleration during walk (ms^2^)	*0.782*	—	—	—	—
Root mean square of the vertical angular velocity during walk (°/s)	*0.530*	—	—	0.469	—
Mean step length (m)	—	*0.903*	—	—	—
Number of steps during walk	—	*–0.900*	—	—	—
Total duration till initial contact with the chair (s)	—	*–0.598*	—	–0.630	—
Total number of steps	—	*–0.771*	—	–0.451	—
Walk duration (s)	–0.405	*–0.786*	—	—	—
*Range of the anteroposterior acceleration during sit-to-walk* (ms^2^)	—	—	*0.823*	—	—
*Range of the mediolateral angular velocity during sit-to-walk* (°/s)	—	—	*0.749*	—	—
Range of the vertical acceleration during sit-to-walk (ms^2^)	—	—	*0.576*	0.422	—
*Root mean square of the mediolateral angular velocity during sit-to-walk* (°/s)	—	—	*0.801*	—	—
Root mean square of the mediolateral angular velocity during turn-to-sit (°/s)	—	—	*0.547*	—	—
Root mean square of the vertical acceleration during sit-to-walk (ms^2^)	—	—	*0.795*	—	—
Mean angular velocity of the 180° turn (°/s)	—	—	—	*0.858*	—
Peak angular velocity of the 180° turn (°/s)	—	—	—	*0.743*	—
Peak angular velocity of the sitting turn (°/s)	—	—	—	*0.827*	—
Turn to sit duration till initial contact with the chair (s)	—	—	—	*–0.823*	—
Range of the mediolateral angular velocity during walk (°/s)	—	—	—	—	*0.845*
Root mean square of the mediolateral angular velocity during walk (°/s)	—	—	—	—	*0.857*

a Italicized values indicate the highest loading for each variable, representing the primary factor association. Only values greater than 0.4 are reported in the table. Variables shown in italics were already normally distributed and did not require logarithmic transformation.

b Not applicable.

### Linear Discriminant Analysis

**Figure 4.**
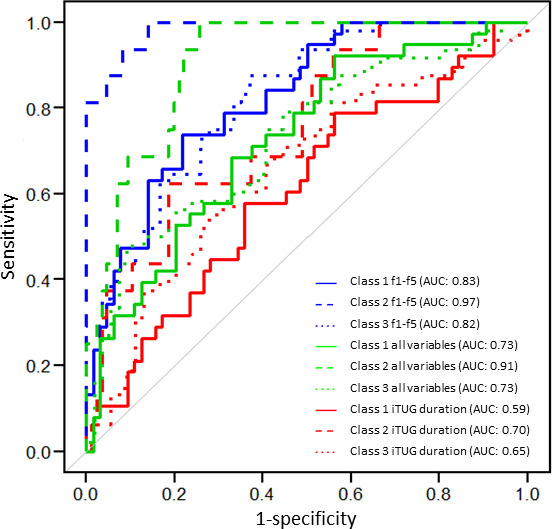
Representation of receiver operating characteristic (ROC) curves. ROC compares the classification performance of instrumented Timed Up and Go (iTUG) duration (variable labeled as iTUG “total duration till initial contact with the chair”; red), factors (f1–f5; blue), and 5 randomly selected variables (green). Sensitivity is plotted against 1–specificity, illustrating the discriminative ability of each variable. The curves demonstrate the ability of different predictors to distinguish between patient diagnoses: total hip arthroplasty (THA; solid lines), total knee arthroplasty (TKA; long dashed lines), and femur fracture (short dashed lines). AUC: area under the curve.

LDA revealed that the first 5 factor scores, corresponding to the factors walking ability, pace or rhythm, sit-to-walk smoothness, turning ability, and mediolateral angular stability, successfully discriminated between the 3 diagnoses with an accuracy of 0.68. These 5 factor scores demonstrated a total AUC of 0.87, with an AUC of 0.83 for THA, 0.97 for TKA, and 0.82 for femur fractures (Figure 4).

The accuracy of the discriminant analysis model using the variable “total duration” was 0.50, with a total AUC of 0.65. In contrast, the model including all iTUG variables with good reliability achieved an accuracy of 0.54 and an AUC of 0.79.

### Measures of Agreement

Table 3 shows the descriptive statistics, test-retest reliability indices, and measurement error parameters for the retained variables (n=25) across 2 sessions (day 1 and day 2). Each variable is accompanied by its mean and SD for both days, reflecting the overall consistency of the measures across time. The SEM provides an estimate of the measurement error attributable to random variations, offering insight into the precision of each variable. Lower SEM values reflect greater measurement precision. The MDC, derived from the SEM, represents the smallest change in a variable that can be interpreted as a true difference beyond measurement error. Larger MDC values indicate greater variability in detecting meaningful changes over time, whereas smaller values suggest higher sensitivity.

**Table 3.** Descriptive statistics and agreement measures of the retained variables (n=25).

Variable	Day 1, mean (SD)		Day 2			ICC^a^			
			Mean (SD)		ICC	95% CI		SEM^b^	MDC^c^ (90%)
Total number of steps	15.298 (4.952)		14.644 (4.110)		0.779	0.689-0.846		2.138	4.973
Mean step length (m)	0.573 (0.192)		0.589 (0.167)		0.775	0.669-0.847		0.085	0.198
Range of the anteroposterior acceleration during sit-to-walk (ms^2^)	9.842 (2.058)		9.702 (2.217)		0.784	0.681-0.853		0.993	2.310
Range of the vertical acceleration during sit-to-walk (ms^2^)	4.724 (2.091)		4.733 (1.812)		0.805	0.712-0.868		0.862	2.005
Root mean square of the vertical acceleration during sit-to-walk (ms^2^)	1.337 (0.512)		1.373 (0.505)		0.882	0.826-0.920		0.174	0.406
Range of the mediolateral angular velocity during sit-to-walk (°/s)	143.058 (53.462)		150.363 (52.583)		0.871	0.808-0.913		19.064	44.351
Root mean square of the mediolateral angular velocity during sit-to-walk (°/s)	52.204 (15.502)		54.974 (15.589)		0.890	0.830-0.927		5.173	12.035
Root mean square of the mediolateral angular velocity during turn-to-sit (°/s)	0.700 (0.305)		0.726 (0.340)		0.482	0.236-0.649		0.232	0.539
Mean angular velocity of the 180° turn (°/s)	66.099 (27.070)		72.208 (29.286)		0.901	0.834-0.938		8.911	20.730
Peak angular velocity of the 180° turn (°/s)	124.330 (42.056)		131.032 (45.224)		0.911	0.863-0.941		13.07	30.405
Peak angular velocity of the sitting turn (°/s)	130.800 (46.552)		140.803 (51.975)		0.889	0.823-0.928		16.489	38.359
Walk duration (s)	9.134 (3.804)		8.181 (3.073)		0.790	0.648-0.869		1.597	3.714
Number of steps during walk	10.019 (4.098)		9.404 (3.287)		0.855	0.783-0.903		1.417	3.296
Range of the anteroposterior acceleration during walk (ms^2^)	5.369 (2.001)		5.697 (2.093)		0.862	0.794-0.908		0.76	1.769
Range of the mediolateral acceleration during walk (ms^2^)	5.868 (2.192)		6.338 (2.704)		0.868	0.797-0.913		0.896	2.085
Range of the vertical acceleration during walk (ms^2^)	6.810 (2.722)		7.349 (2.670)		0.901	0.840-0.937		0.849	1.974
Root mean square of the anteroposterior acceleration during walk (ms^2^)	1.438 (0.490)		1.553 (0.557)		0.810	0.711-0.874		0.23	0.534
Root mean square of the mediolateral acceleration during walk (ms^2^)	0.915 (0.287)		0.982 (0.325)		0.912	0.846-0.946		0.091	0.212
Range of the anteroposterior angular velocity during walk (°/s)	54.028 (19.465)		56.768 (20.359)		0.863	0.797-0.907		7.372	17.150
Range of the mediolateral angular velocity during walk (°/s)	72.046 (27.695)		73.475 (27.123)		0.812	0.723-0.872		11.862	27.595
Root mean square of the anteroposterior angular velocity during walk (°/s)	9.390 (3.202)		9.997 (3.766)		0.888	0.828-0.926		1.172	2.726
Root mean square of the mediolateral angular velocity during walk (°/s)	13.355 (4.248)		14.005 (5.014)		0.901	0.852-0.934		1.46	3.396
Root mean square of the vertical angular velocity during walk (°/s)	20.331 (6.604)		21.725 (6.561)		0.803	0.702-0.868		2.934	6.826
Total duration till initial contact with the chair (s)	17.671 (6.248)		15.838 (5.291)		0.853	0.619-0.929		2.241	5.214
Turn-to-sit duration till initial contact with the chair (s)	4.165 (1.931)		3.683 (1.655)		0.738	0.603-0.826		0.926	2.155

a ICC: intraclass correlation coefficient.

b SEM: SE of measurement.

c MDC: minimal detectable change.

## Discussion

This study demonstrated that, in a sample of heterogeneous orthopedic patients discharged from a rehabilitative ward, only 25 out of the 100 original or exported iTUG variables were both reliable and capable of fitting into a robust assessment model with good indices. EFA on this model identified 5 domains that contribute to motor performance: walking ability, pace or rhythm, sit-to-walk smoothness, turning ability, and mediolateral angular stability. The ability of these factors to differentiate between the 3 orthopedic diagnoses is significantly higher than the total duration of the test. This finding underscores the clinical value of moving beyond single-duration metrics toward a multidimensional, sensor-based assessment framework.

A total of 36 out of the 100 variables derived from the iTUG exhibited excellent test-retest reliability. The fact that only one-third of the variables are reliable is consistent with findings previously reported in a population of elderly patients with femoral fractures [35]. In recent years, several studies have investigated the reliability of the iTUG in different populations [36-39], yet none have conducted a detailed analysis of individual variable reliability in patients with knee or hip replacements.

In our study, “Total Duration till initial contact with the chair” was found to be more reliable than the durations of the individual subphases. The most reliable variables were those related to the walking phase, whereas jerk-based metrics did not reach adequate levels of reliability. This finding is consistent with previous literature suggesting that the limited reliability of jerk-based features may be due to multiple concurrent factors, particularly during postural transitions such as sit-to-stand [39]. As reported by Weiss et al [40], outcome measures like jerk and range, which are highly sensitive to subtle variations and signal noise, may show greater variability and therefore reduced reliability, despite standardized acquisition protocols.

As expected, the measures that can be obtained using a stopwatch—that is, total duration and the gait variables derived from it (ie, gait speed, mean step duration, and cadence)—showed high reliability. It is worth noting that, although these variables can be derived from manual timing, the instrumented test additionally offers the advantage of being operator-independent [41].

Measures of stability, regularity, and coordination exhibited low reliability, likely due to the limited number of steps performed during the iTUG test. It is well established that reliable stability and coordination metrics typically require more than 20 to 25 steps [42,43]. In our sample, participants performed, on average, fewer than 15 steps, highlighting a limitation inherent to the test itself rather than to the measures.

Furthermore, all participants in our orthopedic patient sample used a walking aid, which appears to compromise jerk-related measures by disrupting the natural flow of gait [44]. As a result, only 1 jerk variable during the walking phase demonstrated good reliability.

A recent review has confirmed the reliability of single-point IMUs for gait metric analysis and their potential to support clinical applications, especially when placed over the L3-L4 vertebrae [45]. This approach has been shown to be valid for the iTUG as well [16]. Despite the simplicity of use and the demonstrated validity of using a single IMU placed over the lumbar region, the accuracy of spatiotemporal gait parameter estimation is significantly influenced by sensor placement [46]. For instance, although the average error across multiple strides remained consistent across different sensor positions, significant variations were observed in single-stride errors and variability parameters [47]. Practical considerations, such as ease of execution and time efficiency associated with different IMU configurations, also play an important role. The single-sensor approach offers a clear advantage in terms of usability, as starting data collection is as straightforward as pressing “start” on a smartphone, comparable to using a stopwatch. On the other hand, while adding more sensors can enhance data richness and improve the accuracy of the estimation of some features, it might notably increase the time required to complete the evaluation, and its reliability would still be influenced by sensor placement [47,48]. Therefore, the iTUG test with only a single-sensor setup provides a good balance between sensor count and wearability, which is an optimal tradeoff between accuracy and practicality in real-world applications.

Furthermore, a single sensor for mobility assessment facilitates the transition of clinical evaluations from traditional supervised clinical settings to unsupervised home environments, thereby promoting and enabling continuity of care scenarios where the patient receives integrated, coordinated, and person-centered care, both during acute episodes and in the management of chronic conditions, reducing fragmentation in health care delivery [45].

Previous studies have demonstrated the use of iTUG measures in distinguishing preoperative functional status across various orthopedic conditions [49]. For instance, Bloomfield [50] categorized patients undergoing total knee replacement into moderate-functioning and low-functioning groups based on preoperative test duration. Similarly, Gasparutto et al [51] conducted a biomechanical analysis of the TUG test in total hip replacement patients, comparing functional deficits in each phase of the test in patients with those of a control group. However, these discriminative capabilities within relatively homogeneous orthopedic populations appear to be primarily driven by large differences in total test duration, rather than by the intrinsic discriminative power of the extracted sensor-based variables. In fact, both studies reported differences exceeding the 4-second minimal clinically important difference for orthopedic populations [52], potentially limiting the added value of sensor-based analysis.

In contrast, given the lack of significant differences in total TUG duration among the 3 patient groups, our findings suggest that this measure alone is insufficient to differentiate between groups in a sample of relatively high-functioning individuals. Instead, our iTUG-based model, which incorporates the 5 principal components, provided a significantly superior discriminative ability compared to total duration alone. Specifically, the iTUG model achieved an AUC greater than 0.8, whereas the model based solely on total duration yielded an AUC less than 0.7, indicating limited predictive power.

Overall, our results reinforce the added value and sensitivity of iTUG over the traditional stopwatch-based TUG. At the same time, they show that when sensor technology is not used and only standard variables are considered, the total duration recorded by the iTUG remains comparable to conventional measurements, as recently reported by Dos Santos et al [53]. This underscores the importance of integrating sensor-based analytics to refine mobility assessments and optimize rehabilitation strategies, ultimately improving clinical decision-making and patient care.

Although the LDA conducted in this study demonstrated good discriminant ability among groups (mean overall accuracy 0.68, SD 0.14), more advanced predictive models, such as machine learning algorithms, could perform even better. Machine learning approaches have already been applied to iTUG assessments in various populations and for different purposes, including evaluating levodopa responsiveness in individuals with Parkinson disease [54] and predicting falls across different pathologies [1]. In this perspective, the present study proposes a reliability-driven and interpretable framework for variable selection and dimensionality reduction, which may provide a preliminary layer of feature selection for the development of predictive models not only in orthopedic rehabilitation but also in other populations characterized by mobility impairments, such as neurological disorders. While the discriminative patterns identified here are specific to postsurgical orthopedic patients, the latent mobility domains derived from the iTUG (eg, walking ability, turning, sit-to-walk smoothness, and mediolateral stability) consist of fundamental components of functional mobility, which are also affected in other conditions, including neurological disorders.

This study has several clinical implications. First, the use of multidimensional, sensor-derived metrics, particularly those related to walking dynamics, offers a standardized and operator-independent approach to assessing mobility in orthopedic patients. This supports consistent tracking of rehabilitation progress across settings. Second, the factor structure identified through EFA provides clinicians with interpretable domains (eg, sit-to-walk smoothness and mediolateral angular stability) with strong discriminative performance (AUC>0.8) that can inform targeted therapy planning beyond total time. Third, the iTUG model’s ability to discriminate between patients with similar test durations suggests that it can uncover latent performance differences that might otherwise remain undetected, especially in high-functioning individuals.

Selected variables showed stable and reproducible measurements in the present dataset. This suggests their potential suitability not only for cross-sectional group differentiation but also for longitudinal monitoring, where consistency of measurement is required to detect true change beyond measurement noise. From a clinical perspective, this capability is particularly relevant for stratifying and monitoring patients within the same orthopedic condition, such as those undergoing THA or TKA. The assessment and selection of sensor-based features represent an initial step toward the development of an evaluation model aimed at quantifying motor impairments that may distinguish the categories and reflect rehabilitation targets. The underlying objective is to capture functional dimensions that could inform treatment decisions; accordingly, these variables may serve as candidate outcome measures to track patient progress, evaluate treatment effectiveness, and support early adjustment of rehabilitation strategies. They may also contribute to finer personalization within each category by highlighting individual functional profiles.

Finally, once a reliable and clinically meaningful feature set is identified, the same variables could provide a basis for additional predictive models, such as fall-risk estimation or forecasting functional decline. However, these findings should be considered exploratory. Validation in larger and more diverse cohorts will be necessary to assess the robustness, generalizability, and actual performance of both the assessment and predictive models and to determine their potential clinical use. Future work will therefore focus on increasing the sample size and conducting within-diagnosis analyses to identify distinct functional recovery profiles, enabling the distinction between patients who are recovering as expected and those showing subtle but clinically meaningful deviations from typical recovery trajectories. Such early identification may support timely, personalized rehabilitation adjustments before functional decline or plateau becomes evident using conventional time-based measures.

Moreover, the feasibility of a single-sensor setup reinforces the iTUG’s potential for remote monitoring and continuity of care postdischarge. Reliable domains such as walking ability and pace or rhythm may represent valuable longitudinal indicators of recovery and could be integrated into digital tools for monitoring rehabilitation outcomes.

In conclusion, this study confirms the reliability of selected iTUG variables in orthopedic patients and reinforces their clinical value for a multidimensional assessment of motor performance. Factor analysis identified 5 meaningful components capable of distinguishing between common orthopedic conditions, addressing the limitations of relying solely on total test duration. These findings support the hypothesis that the development of predictive models and clinical decision support tools based on sensor-based functional tests, such as the iTUG, has the potential to improve clinical decision-making and optimize rehabilitation interventions.

## Supplementary material

10.2196/82632 Multimedia Appendix 1 The 100 parameters extracted from the instrumented Timed Up and Go (iTUG) test, categorized according to the specific functional phases: sit-to-walk, walking, turning, and turn-to-sit.

## Abbreviations

AUC: area under the curve
EFA: exploratory factor analysis
ICC: intraclass correlation coefficient
IMU: inertial measurement unit
iTUG: instrumented Timed Up and Go
KMO: Kaiser-Meyer-Olkin
LDA: linear discriminant analysis
MDC: minimum detectable change
PCA: principal component analysis
ROC: receiver operating characteristic
SEM: SE of measurement
THA: total hip arthroplasty
TKA: total knee arthroplasty
TUG: Timed Up and Go
