# Supplementary material for: Reliability and Discriminant Ability of an Instrumented Timed Up and Go Test in People With Postsurgical Orthopedic Conditions: Quantitative Study
Source: JMIR Rehabil Assist Technol. 2026 Apr 1;13:e82632. doi: 10.2196/82632 (PMC13043016; doi:10.2196/82632)
Supplement: Multimedia Appendix 1 [file rehab-v13-e82632-s001.pdf]

Supplementary Materials 1

Validated algorithms [1,2,3] were used to automatically split the TUG test into the sit-to-walk, walking, turning and turn-and-sit phases and to calculate 100 parameters.

ITUG parameters from the Sit-To-Walk phase (n = 21).

**Variables**
Sit-to-Walk Duration [s]
Range of Anterior-Posterior Acceleration during the Sit-to-Walk Transition [m/s^2]
Range of Medio-Lateral Acceleration during the Sit-to-Walk Transition [m/s^2]
Range of Vertical Acceleration during the Sit-to-Walk Transition [m/s^2]
Root Mean Square of Anterior-Posterior Acceleration during the Sit-to-Walk Transition [m/s^2]
Root Mean Square of Medio-Lateral Acceleration during the Sit-to-Walk Transition [m/s^2]
Root Mean Square of Vertical Acceleration during the Sit-to-Walk Transition [m/s^2]
Jerk Score for Anterior-Posterior Acceleration during the Sit-to-Walk Transition [m]
Jerk Score for Medio-Lateral Acceleration during the Sit-to-Walk Transition [m]
Jerk Score for Vertical Acceleration during the Sit-to-Walk Transition [m]
Range of Angular Velocity about the Anterior-Posterior Axis during the Sit-to-Walk Transition [°/s]
Range of Angular Velocity about the Medio-Lateral Axis during the Sit-to-Walk Transition [°/s]
Range of Angular Velocity about the Vertical Axis during the Sit-to-Walk Transition [°/s]
Root Mean Square of Angular Velocity about the Anterior-Posterior Axis during the Sit-to-Walk Transition [°/s]
Root Mean Square of Angular Velocity about the Medio-Lateral Axis during the Sit-to-Walk Transition [°/s]
Root Mean Square of Angular Velocity about the Vertical Axis during the Sit-to-Walk Transition [°/s]
Normalised Jerk Score for Angular Velocity about the Anterior-Posterior Axis during the Sit-to-Walk Transition
Normalised Jerk Score for Angular Velocity about the Medio-Lateral Axis during the Sit-to-Walk Transition
Normalised Jerk Score for Angular Velocity about the Vertical Axis during the Sit-to-Walk Transition
Power of the Vertical Push Off in the Sit-to-Walk Transition [Nm]
Sitting Turn Duration [s]

ITUG parameters from the Walk phase (n = 49).

Variables
Walk Duration [s]
Gait Speed [m/s]
Number of Steps in the Walk Phase (not including turns)
Mean Step Length [m]
Mean Step Duration [s]
Step Duration Standard Deviation [s]
Step Duration Coefficient of Variation [%]
Step Regularity in the Anterior-Posterior Direction [%]
Step Regularity in the Medio-Lateral Direction [%]
Step Regularity in the Vertical Direction [%]
Stride Regularity in the Anterior-Posterior Direction [%]
Stride Regularity in the Medio-Lateral Direction [%]
Stride Regularity in the Vertical Direction [%]
Cadence [steps/min]
Gait Symmetry in the Anterior-Posterior Direction
Gait Symmetry in the Medio-Lateral Direction
Gait Symmetry in the Vertical Direction
Range of Anterior-Posterior Acceleration during the Walk Phase [m/s^2]
Range of Medio-Lateral Acceleration during the Walk Phase [m/s^2]
Range of Vertical Acceleration during the Walk Phase [m/s^2]
Root Mean Square of Anterior-Posterior Acceleration during the Walk Phase [m/s^2]
Root Mean Square of Medio-Lateral Acceleration during the Walk Phase [m/s^2]
Root Mean Square of Vertical Acceleration during the Walk Phase [m/s^2]
Range of Angular Velocity about the Anterior-Posterior Axis during the Walk Phase [°/s]
Range of Angular Velocity about the Medio-Lateral Axis during the Walk Phase [°/s]
Range of Angular Velocity about the Vertical Axis during the Walk Phase [°/s]
Root Mean Square of Angular Velocity about the Anterior-Posterior Axis during the Walk Phase [°/s]
Root Mean Square of Angular Velocity about the Medio-Lateral Axis during the Walk Phase [°/s]
Root Mean Square of Angular Velocity about the Vertical Axis during the Walk Phase [°/s]

Jerk Ratio for the Antero-Posterior Direction in the Walk Phase
Total Duration [s]
Total Duration till initial contact with the chair [s]
Walk Duration including the 180° Turn [s]
Time-Normalized Jerk Score of the Antero-Posterior Acceleration during Walk [m]
Time-Normalized Jerk Score of the Medio-Lateral Acceleration during Walk [m]
Time-Normalized Jerk Score of the Vertical Acceleration during Walk [m]
Total Number of Steps
Harmonic Ratio in the Antero-Posterior direction
Harmonic Ratio in the Medio-Lateral direction
Harmonic Ratio in the Vertical direction
Mean of Phase Differences [°]
Standard Deviation of Phase Differences [°]
Mean Phase [°]
Standard Deviation of Phase [°]
Coefficient of Variation of Phase [%]
Phase Coordination Index [%]
Walk/Turn Ratio Outward
Walk/Turn Ratio Return
Walk/Turn Ratio Overall

ITUG parameters from the Turning phase (n = 6).

**Variables**
180° Turn Duration [s]
Turning Angle of the 180° Turn [°]
Mean Angular Velocity of the 180° Turn [°/s]
Peak Angular Velocity of the 180° Turn [°/s]
Normalised Jerk Score for the 180° Turn
Number of Steps in the 180° Turn

ITUG parameters from the Turn-and-Sit phase (n = 24).

**Variables**
Turn Duration in the Turn-and-Sit Phase [s]
Turn to Sit Duration till initial contact with the chair [s]
Range of Anterior-Posterior Acceleration during the Turn-to-Sit Transition [m/s^2]
Range of Medio-Lateral Acceleration during the Turn-to-Sit Transition [m/s^2]
Range of Vertical Acceleration during the Turn-to-Sit Transition [m/s^2]
Root Mean Square of Anterior-Posterior Acceleration during the Turn-to-Sit Transition [m/s^2]
Root Mean Square of Medio-Lateral Acceleration during the Turn-to-Sit Transition [m/s^2]
Root Mean Square of Vertical Acceleration during the Turn-to-Sit Transition [m/s^2]
Jerk Score for Anterior-Posterior Acceleration during the Turn-to-Sit Transition [m]
Jerk Score for Medio-Lateral Acceleration during the Turn-to-Sit Transition [m]
Jerk Score for Vertical Acceleration during the Turn-to-Sit Transition [m]
Range of Angular Velocity about the Anterior-Posterior Axis during the Turn-to-Sit Transition [°/s]
Range of Angular Velocity about the Medio-Lateral Axis during the Turn-to-Sit Transition [°/s]
Range of Angular Velocity about the Vertical Axis during the Turn-to-Sit Transition [°/s]
Root Mean Square of Angular Velocity about the Anterior-Posterior Axis during the Turn-to-Sit Transition [°/s]
Root Mean Square of Angular Velocity about the Medio-Lateral Axis during the Turn-to-Sit Transition [°/s]
Root Mean Square of Angular Velocity about the Vertical Axis during the Turn-to-Sit Transition [°/s]
Normalised Jerk Score for Angular Velocity about the Anterior-Posterior Axis during the Turn-to-Sit Transition
Normalised Jerk Score for Angular Velocity about the Medio-Lateral Axis during the Turn-to-Sit Transition
Normalised Jerk Score for Angular Velocity about the Vertical Axis during the Turn-to-Sit Transition
Turning Angle of the Turn-and-Sit Phase [°]
Mean Angular Velocity of the Turn-and-Sit Phase [°/s]
Peak Angular Velocity of the Turn-and-Sit Phase [°/s]
Normalized Jerk Score of the Angular Velocity of the Sitting Turn

REFERENCES

1. Mellone S, Tacconi C, Chiari L. Validity of a Smartphone-based instrumented Timed Up and Go. Gait Posture. 2012;36(1): 163-165.

2. Mellone S. Movement Analysis by Mean of Interial Sensors: From Bench to Bedside. PhD Thesis, Alma Mater Studiorum Università di Bologna, Bologna, Italy; 2013.

3. Coni A, Mellone S, Colpo M, Guralnik JM, Patel K V, Bandinelli S, et al. An Exploratory Factor Analysis of Sensor-Based Physical Capability Assessment. Sensors. 2019;19(10): 2227.
